# Health Profile of Textile Workers in Surat

**DOI:** 10.4103/0970-0218.43241

**Published:** 2008-10

**Authors:** RK Bansal, JD Sodvadiya, PJ Bharodiya, RV Sanghani, DR Shah

**Affiliations:** Department of Community Medicine, Surat Municipal Institute of Medical Education and Research, Surat 395 010, Gujarat, India. E-mail: drrkbansal@gmail.com

Sir,

This study reports the findings from interviews conducted among 100 textile workers from Surat to elicit information pertaining to their health profile. The findings revealed that almost all of these men are migrant workers from UP, Bihar, West Bengal, or Orissa.(1) Almost all of them are married (96%) and are living as forced bachelors in Surat as their employers are providing only single, shared accommodations for them and private housing rentals are beyond their paying capacity. What is of concern is the fact that more that half of them are visiting commercial sex workers (CSWs) to fulfil their sexual needs, as they are able to visit their families for only very limited periods mainly during Diwali when the textile mills are closed for a week.

Also equally alarming is the issue of irregular usage of condoms with the CSWs, though the situation is far better than for other risk groups.(2) Among 53 men who visited CSWs, 48 (90.6%) used condoms regularly. Looking to the high seropositivity among CSWs and the possibility of these workers acting as a conduit for further transmission to the general population,(3) the issue of irregular condom usage needs to be urgently rectified in the context of the contraction of sexually transmitted infections (STIs), including HIV and AIDS. The comparatively higher use of condoms observed in the study as compared with that reported in other areas is due to the extensive outreach activities undertaken by the Partnership for Sexual Health Project undertaken by the Surat Municipal Seva Sadan. Factors compounding their risk-taking sexual behaviour include their habits of regular consumption of alcohol (32%) and tobacco (86%), relatively easier earnings, and disrupting of their social structure. It is well-known that factors such as alcohol intoxication promotes the adoption of risky behaviours and impairs judgement in the use of condoms. What is equally conspicuous is their absolute dependence on private medical care providers as they are not well acquainted with the health services provided to routine residents because they are migrants. It is important to note that many of these private providers are not qualified medical practitioners and are unable to treat and diagnose STIs adequately.

This study highlights the urgent need for still greater emphasis on health educational programs on safe sex, STIs, and the prevention of alcohol use among these migrant workers to decrease their risk taking behaviour and vulnerability to STIs including HIV and AIDS. Equally important is the issue of ensuring that these workers are covered within the ambit of the urban public healthcare services so that they can receive proper medical care for STIs and necessary counselling. The employers and the government need to understand the gravity of the impending threat of epidemics such as HIV/AIDs and cardiovascular problems. The contemporary concerns call for flexible approaches and abandonment of the traditional dogmatic approaches.

Some of the other factors influencing their health status relate to their tobacco use, fast food consumption (96%), absence of regular exercise (89%), inadequate sleep and rest (48%), and inadequate provision of industrial health care services. Also, their prolonged absence from home negatively impacts the health care seeking patterns of their family members residing in their native places. Such issues also merit attention. Eventually, on a long-term basis, there is a need for provision of suitable family status accommodations for such workers as this step would solve many of the problems mentioned in this study.

## References

[1] Halli SS, Blanchard J, Satihal DG, Moses S (2007). Migration and HIV transmission in rural South India: An ethnographic study. Cult Health Sex.

[2] Bansal RK (1995). Truck drivers and risk of STDs including HIV. Indian J Community Med.

[3] Faisel A, Cleland J (2006). Migrant men: A priority for HIV control in Pakistan?. Sex Transm Infect.

